# Assessing the sensitisation hazard of microbial pesticides: potential value of New Approach Methodologies (NAMs) to overcome current challenges

**DOI:** 10.1007/s00204-025-04149-2

**Published:** 2025-08-27

**Authors:** Daniela Morais Leme, Cynthia Bomfim Pestana, Elaine F. Kenny, Sabrina Feustel, Philip Marx-Stoelting, Emanuela Corsini

**Affiliations:** 1 Department of Genetics, Federal University of Paraná (UFPR), Curitiba, PR Brazil; 2 German Federal Institute for Risk Assessment (BfR), Berlin, Germany; 3 Laboratory of Toxicology, Department of Pharmacological and Biomolecular Sciences, Rodolfo Paoletti’, Università Degli Studi Di Milano, Via Balzaretti 9, 20133 Milan, Italy

**Keywords:** Microbial pesticides, Microorganisms, Skin sensitisation, Respiratory sensitisation, New approach methodologies

## Abstract

Over the past years, the use of chemical pesticides has increased considerably worldwide, and concerns about human health and the environment have stimulated the development of safer alternatives. Biopesticides, including those with microorganisms as active substances, *i.e.* microbial pesticides, appear to be specific in action, easily sourced without the need for expensive chemicals, and environmentally sustainable with no residual effects. As such, they are seen as a viable alternative to synthetic pesticides. As with any other plant protection product, microbial pesticides are subjected to regulatory approval before marketing, and sensitisation, either via the dermal and/or inhalation routes, is one of the data requirements that have to be addressed in this process. The biological mechanisms underlying potential concerns related to sensitisation properties of microbial pesticides are reviewed in this article. Mechanistic knowledge was utilised to understand the potential limitations of current testing strategies for assessing sensitisation hazards, primarily defined by chemicals, as well as to demonstrate the potential value of New Approach Methodologies (NAMs) in this process. However, evaluating the sensitisation properties of microbial pesticides requires several protocol adaptations to achieve adequate confidence levels for alternative methods, narrow their applicability domain, and provide potency information on this endpoint. The technical limitations and difficulties in interpreting the results of current testing methods applied to microbial pesticides have long been recognised and are discussed in this article to better drive propositions of NAM-based strategies for microbial pesticides.

## Introduction

Currently, there is an increasing global demand for the utilisation of biopesticides by the agrochemical industry to reduce potential negative effects on human health and the environment perceived to be associated with traditional synthetic pesticides (Akmukhanova et al. 2023). Biopesticides include biochemical compounds (*e.g.* plant extracts/botanicals, pheromones and semiochemicals), microbial pesticides (bacteria, algae, protozoa viruses and fungi) and other modes of action such as RNA interference (RNAi), genetically modified plants or the use of invertebrates (insects and nematodes). Microbial pesticides are the most widely used and studied class of biopesticides. They antagonise plant pathogens and pests through various mechanisms, including the production of toxins, the secretion of enzymes, the release of volatile compounds, direct colonisation or consumption of the host, the induction of resistance in crop plants and competition for nutrients and space (Wend et al. 2024). Unlike synthetic pesticides, microbial pesticides can be highly specific to a target pest, can be easily sourced without the need for expensive chemicals, and are considered environmentally sustainable without residual effects (Ayilara et al. 2023). However, in addition to their ability to reproduce and be metabolically active in situ after application, microorganisms have the potential to be pathogenic and/or infective and to transfer genes that confer antimicrobial resistance properties (AMR). There is also a risk of sensitisation in operators, workers and bystanders, as well as the potential production of metabolites of concern (OECD 2023a).

In 2010, the European Food Safety Authority (EFSA) investigated the potential of microorganisms, microbial products and enzymes to induce respiratory sensitisation when used as food and feed additives, by conducting a systematic literature search examining the link between microorganisms and respiratory sensitisation. They reviewed the field of microorganisms and occupational health, surveying existing regulations on the subject. They concluded that microorganisms should be assumed a priori to be respiratory sensitisers unless convincing evidence to the contrary was provided (Martel et al. 2010).

The Microbial Pesticide Test Guidelines (OPPTS Series 885) are one of a series of test guidelines developed by the United States Environmental Protection Agency (EPA) for testing microbial pesticides to meet the requirements of the Federal Insecticide, Fungicide, and Rodenticide Act (FIFRA). Besides specifically addressing human safety for diverse microorganisms, the required guideline studies were designed to evaluate the potential pathogenicity and infectivity of the microbe through various routes of administration. In addition, information on sensitisation is also needed, according to the data requirements for both microbial active substances and products (OECD 2023a).

In the European Union (EU), pesticidal substances are categorised either as plant protection products (PPPs), which are used on crops, or biocides, which include disinfectants, preservatives and non-crop pest control agents. PPPs (Regulation (EU) 1107/2009 with the respective data requirements for active substances and products and uniform principles in their amended versions) and biocides (Regulation No. 528/2012) are regulated by different legislations. However, both regulatory frameworks require an assessment of potentially sensitising properties for the active substances as well as the marketed products, independent of the tonnage level (Daniel et al. 2018). The efforts to reduce, replace and refine the use of animal testing, along with the recent increase in New Approach Methodologies (NAMs), have highlighted the need to adjust existing guideline studies to meet the requirements of testing microbial pesticides. According to Regulation (EU) 283/2013, part B (amended under Regulation (EU) 2022/1439), tests on vertebrate animals should be undertaken only where no other validated methods are available (Wend et al. 2024).

The 7th biopesticides steering group seminar on the sensitisation potential of microorganisms reported that dossiers of microbial pesticides either present no studies on sensitisation or present study protocols routinely used for testing the skin sensitisation potential of chemical substances (Buehler, GPMT and LLNA), which are not validated for microbial pesticides, yielding results that are difficult to interpret. Consequently, information on the sensitisation potential of species used for plant protection relies on published literature on the strain under evaluation or closely related species. Considering that there are no validated test methods for the sensitisation of microorganisms and the difficulty in negating sensitisation hazards without appropriate testing, EU regulators face the situation of requiring specific labelling with a warning phrase on the PPP containing microorganisms as the active substances. The precautionary sentence, ‘Microorganisms may have the potential to provoke sensitising reactions’, was established under Regulation (EU) No 283/2013 and the Uniform Principles from Regulation (EU) No 546/2011. This statement does not imply that microorganisms are inherently sensitisers, but rather that they have the potential to cause sensitisation. Negating the hazard is only possible if the applicant provides data from validated test methods demonstrating that the microorganisms do not cause sensitisation.

The request for personal protective equipment (PPE) for both operators and re-entry workers is also affected by the lack of adequate test methods for microorganisms, as intrinsic toxicological hazard data cannot be used as the basis for PPE assignment, unlike in chemical PPPs (Lichtenberg et al. 2015). Consequently, for PPPs containing microorganisms, the default PPE requirement involves gloves, protective clothing, and sturdy footwear when handling concentrated microbial products during mixing and loading. A particle-filtering mask may also be required when handling powdered or dust formulations. However, there are no harmonised mandatory PPE instructions for operators across all EU Member States (Lichtenberg et al. 2015).

Given the urgent need for the approval of low-risk active substances in light of the EU Chemical Strategy for Sustainability’s requirements, the application of NAMs in the field of biopesticides appears promising, considering the diverse endpoints addressed by various NAMs and respective decision strategies (OECD 2023a). This review article aims to present comprehensive mechanistic insights into the sensitisation properties of microorganisms via dermal and inhalation routes and to compare them with the mechanisms underlying current testing approaches for chemical sensitisation. By deepening our understanding of these processes, this review aims to pave the way for the development of NAMs that can enhance sensitisation assessments of microbial pesticides, one of the most challenging endpoints in this field.

## Sensitisation: allergic properties

Hypersensitivity can be defined as the excessive humoral or cellular immune response to an antigen, which can lead to tissue damage. Allergic sensitisation is characterised by a tolerance breakdown upon initial exposure to the allergen, followed by subsequent immunological imprinting (Thá et al. 2021; Wang et al. 2023). The induction of an adaptive immune response to a respective allergen in susceptible individuals defines skin and respiratory sensitisation. In both cases, the adaptive immune response is regulated by T cells; however, T cell subpopulations activated typically differ between skin and respiratory sensitisation. Whilst skin sensitisers, particularly chemicals, primarily induce T helper 1 (Th1)-type reactions, respiratory sensitisers preferentially elicit T helper 2 (Th2) response (Hargitai et al. 2024; Thá et al. 2021). Nevertheless, Th2-mediated response can also occur in the skin, particularly in the presence of protein allergens in food (Janssen et al. 2024). Allergic contact dermatitis (ACD), allergic rhinitis and asthma are the most common clinical manifestations of skin and respiratory sensitisation, respectively (Thá et al. 2021). The following sections outline the mechanisms underlying skin and respiratory sensitisation in the context of chemicals and microbial pesticides (*i.e.* microorganisms as the active substances).

### Testing the skin and respiratory sensitisation hazards of chemicals

Skin sensitisation has historically been evaluated using laboratory animals, primarily by observing erythema and oedema skin reactions. The original protocols to evaluate skin sensitisation (OECD TG 406) described two types of tests: the guinea pig maximisation test (GPMT), which uses an adjuvant (Freund’s Complete Adjuvant – FCA), and the non-adjuvanted Buehler test, which has been shown to be less sensitive than the GPMT, but also less invasive (OECD 2022). However, both the GPMT and Buehler provide limited information on quantitative potency depending on the dose level selection (OECD 2022), and to overcome this limitation, the Local Lymph Node Assay (LLNA) (OECD TG 429, OECD TG 442A, OECD TG 442B), a murine test, was developed. LLNA assesses the induction phase exclusively and provides an advantage over guinea pig tests in terms of animal welfare, along with an objective measurement of the induction phase of skin sensitisation. OECD TG 406 (OECD 2022) and 442B have been recently updated (OECD 2024); they draw attention to alternative methods for skin sensitisation and recommend, for animal welfare reasons, that animal tests (Buehler, GPMT, and LLNA) should be conducted only as a last resort, if justified, for instance, when other skin sensitisation test methods are not applicable.

Today, several non-animal methods are available to assess skin sensitisation of chemicals at the regulatory level. Generally, this assessment has been defined for low-molecular-weight (LMW) chemicals based on key biological events that ultimately result in ACD (the adverse outcome). Briefly, LMW chemical sensitisers following contact and absorption into the skin undergo the haptenation process (Molecular Initiation Event—MIE, Key Event 1—KE1), leading to the activation of keratinocytes (KCs) (KE2) and dendritic cells (DCs) (KE3). Activated DCs can then migrate to the draining lymph node to coordinate organ-level responses by presenting the antigen to naïve T cells, leading to T-cell proliferation and differentiation into allergen-specific memory T cells (KE4). The acquisition of sensitivity is the key physiological response, and upon subsequent contact with the same allergen, an allergic reaction can be elicited (elicitation phase), inducing a skin inflammatory reaction (organism response) (OECD 2012).

These molecular and cellular events were utilised to develop the Adverse Outcome Pathway (AOP) for skin sensitisation (OECD 2012), which is currently used by the OECD 497 Defined Approaches (DAs) to evaluate the skin sensitisation hazard potential of chemicals (OECD 2023b). The OECD 497 DAs are entirely based on NAMs and can provide information equivalent to that in animal studies (*i.e.* hazard identification and/or potency categorisation) for LMW chemicals; UVCBs (unknown or variable composition, complex reaction products, or biological materials), for instance, may not be within the applicability domain for validated OECD test methods and may yield inconclusive DA predictions (de Souza et al. 2024; OECD 2023b).

For respiratory sensitisation, mechanistic knowledge is mostly related to protein respiratory allergy (*i.e.* caused by high-molecular-weight (HMW) substances, such as pollen and dust mite excreta) (Thá et al. 2021), and IgE antibodies play a central role in this biological process (Kimber et al. 2018). After exposure to a protein allergen, airway epithelial cells express proinflammatory cytokines, such as IL-25, IL-33, and thymic stromal lymphopoietin (TSLP), thereby activating lung-resident DCs, which produce Th2-type cytokines and recruit eosinophils and neutrophils. Mature lung DCs migrate to draining lymph nodes and induce CD4^+^ T cells to differentiate into Th2 cells. These cells also produce Th2-type cytokines to induce the differentiation of B cells into antigen-specific IgE-producing cells. The allergen-specific IgEs interact with local tissue mast cells and basophils, and following subsequent exposure to the same allergen, mast cell activation and degranulation occur, resulting in the release of several inflammatory mediators. Eosinophil infiltration is also induced by Th2-type cytokines derived from memory Th2 cells (Kimber et al. 2018; Thá et al. 2021).

In contrast to skin sensitisation, there is no widely accepted AOP for respiratory sensitisation, although some have been proposed. In addition, to date, neither validated in vivo test methods nor NAMs are available to assess this endpoint at the regulatory level (Kimber et al. 2018; Martel et al. 2010; Thá et al. 2021; Zeller et al. 2018). Thus, respiratory sensitisation is currently deduced from occupational evidence.

Test methods used for skin sensitisation hazard assessment have been employed as a surrogate for respiratory sensitisation, as similar KEs are also expected to occur in respiratory sensitisation (Bassan et al. 2021). For LMW chemicals, considering the shared AOP (AOP: 39; AOP: 40), chemicals that fail to elicit positive responses in the in vitro methods, validated for skin sensitisers, should be regarded as lacking not only skin sensitising activity but also the potential to induce sensitisation of the respiratory tract. If positive, further investigations are needed to discriminate skin from respiratory sensitisers. However, whether a similar strategy can be applied to microbial pesticides needs to be proven.

Efforts to develop NAMs for respiratory sensitisation, capable of discriminating them from skin sensitisers, irritants, and asthmagens, and achieving regulatory acceptance are growing due to the importance of identifying respiratory sensitisers to prevent cases of work-related allergies (*e.g.* occupational asthma) (Pemberton and Kimber 2021). For example, a respiratory sensitisation AOP for LMW chemicals was proposed by Sullivan et al. (Sullivan et al. 2017), and it is publicly available at AOP-Wiki (AOP: 39). This AOP has been instrumental in driving the advancements of NAMs for identifying chemical respiratory allergens (Arts 2020; Thá et al. 2021). Examples of NAMs showing high specific prediction capacity include the in silico tool Derek Nexus and the GARD® in vitro air method (Thá et al. 2021). Recently, the Universal Immune System Simulator, a state-of-the-art platform for simulating the dynamics of the immune system, has been shown to closely replicate trimellitic anhydride-induced immune reactions, primarily showing Th2-type responses with cytokine patterns typical of allergies, demonstrating its ability to accurately predict the entities and mechanisms involved in the immune response to a specific chemical, as opposed to 2,4-dinitrochlorobenzene (DNCB) that triggers a different type of response (Crispino et al. 2023).

### Mechanisms of sensitisation potentially triggered by microbial pesticides

The skin and respiratory tract are key contact sites for microbial pesticides, particularly for operators and workers (OECD 2023a). Microorganisms are also considered contributing factors in the pathogenesis of allergic diseases, as they are capable of altering the human microbiota and inducing immune responses and inflammatory reactions that may facilitate the development of allergy (Spök et al. 2018; Wang et al. 2023). For some microorganisms currently approved in the EU, positive findings on sensitisation (*i.e.* IgE measured in human serum samples from Danish greenhouse workers) have been reported (Doekes et al. 2004). These reactions are considered rare for bacteria and yeasts and more frequent for fungal species as their sensitisation is primarily attributed to the glycoproteins, proteins, or secondary metabolites they secrete (Wend et al. 2024), rather than to the entire microorganism, which, in the body, will trigger the innate host defence. Viruses are much less likely to cause sensitisation since they lack their own metabolism and, therefore, do not synthesise secondary metabolites (Paege et al. 2024). Also, sensitisation mediated by microorganisms depends on the route and frequency of exposure.

Significant challenges exist in assessing the sensitisation potential of microorganisms due to their unique features compared with those of conventional chemicals (Loprieno 2012; Martel et al. 2010; Spök et al. 2018). Depending on the nature of the microbial pesticides, the sensitisation mechanisms, especially in the skin, may also differ from those of LMW chemicals, for which regulated and well-accepted DAs are available, as outlined above (OECD 2023c).

In principle, dermal sensitisation to microorganisms (*i.e.* their products, such as proteins and metabolites) seems unlikely, as they are unable to penetrate an intact skin/epithelial barrier and access the viable epidermis (Basketter and Kimber 2022; Loprieno 2012). The epithelial barrier, and hence skin homeostasis, is maintained through robust communication between skin commensal microbes and skin cells. Typically, HMW molecules, including proteins, cannot penetrate the skin. However, recent findings have shown that proteins can penetrate damaged or ruptured skin, presenting a potential risk for triggering an allergic response. In addition, certain microorganisms can directly or indirectly affect the skin barrier (Lunjani et al. 2021). For example, the pathogenic bacteria *Staphylococcus aureus* can impair the epidermal barrier by secreting exotoxins (*e.g.* α-toxin) and extracellular proteases during skin colonisation, and its overabundance can lead to dysbiosis. This dysregulation is associated with the pathogenesis of atopic dermatitis (AD) (Lunjani et al. 2021). Barrier-disrupted skin, a condition typically observed in AD, has the potential to induce cytokine responses and increase the density of antigen-presenting cells (APCs) in the skin, resulting in a proallergic state. Although this remains a controversial topic, AD is considered a significant risk factor for the subsequent development of ACD (Johnson et al. 2022). Thus, whilst microorganisms may not be skin sensitisers on their own, they can enhance sensitisation to other allergens.

Upon breach of the epithelial barrier, specific microbial components initiate a signalling cascade mediated by the innate immune system via the Toll-like receptors (TLRs) or NOD-like receptors (NLRs), two families of receptors that recognise pathogen-associated molecular patterns (PAMPs) (Kaplan et al. 2012; Martinon et al. 2009). For example, the recognition of lipopolysaccharide (LPS) (a cell wall component of gram-negative bacteria, endotoxin) is mediated by TLR4. The activation of this receptor results in the production of proinflammatory cytokines, such as IL-6 and TNF-α, as well as the secretion of type I interferons (IFN) (Kaplan et al. 2012; O’Neill and Bowie 2007), which facilitates the activation of a specific immune response.

The activation of NLRs (via recognition of components such as peptidoglycan, flagellin and fungal hyphae) results in the release of IL-1β and IL-18 (Chou et al. 2023), two proinflammatory cytokines required for hapten-induced DC maturation and migration from the skin to the local lymph node. As haptens induce the production of endogenous ligands that activate TLRs and induce innate immunity via NLRs, they are considered early immune events of ACD induction (Kaplan et al. 2012).

In the respiratory tract, sensitisation to microorganisms is primarily associated with proteins and enzymes exhibiting specific characteristics that facilitate the induction of immune responses, leading to allergic sensitisation (Basketter and Kimber 2022). The allergic response to these proteins in the respiratory tract is typically a type I hypersensitivity reaction, *i.e.* IgE antibody-mediated allergy, as described above in Sect. “Testing the skin and respiratory sensitisation hazards of chemicals”.

Fungal-mediated airway diseases are examples of respiratory sensitisation caused by microorganisms that have a global health impact. These diseases include asthma (affecting more than 300 million people worldwide), severe asthma with fungal sensitisation (SAFS), allergic bronchopulmonary aspergillosis (ABPA) and allergic fungal rhinosinusitis (AFRS) (Furlong-Silva and Cook 2022). *Aspergillus*, *Cryptococcus* and *Pneumocystis*, spp. are the major inducers of these lung diseases (Li et al. 2019).

Through their ability to sense fungi via the Dectin family of receptors, macrophages and dendritic cells (DCs) in the lung play a crucial role in initiating the immune response and removing spores from the lung (Heung et al. 2023). However, they are also central to the development of respiratory disease caused by fungi. The factors leading these cells to switch from immune surveillance and pathogen neutralisation to initiators of an allergic response are unclear to date.

Concerns of systemic sensitisation are raised in food allergies because the organ interplay in the gut–immune–skin axis is related to the process of inducing protein allergy mediated by IgE antibody mechanisms (Janssen et al. 2024). However, for enzymes used in laundry and cleaning products, skin appears to be ineffective in driving systemic sensitisation, specifically sensitisation of the respiratory tract (Basketter and Kimber 2022). The possibility of biopesticide residues in food triggering systemic sensitisation remains to be demonstrated.

### The potential role of NAMs in assessing sensitisation to microbial pesticides

Whilst NAMs may hold significant promise for assessing microorganisms, several critical factors need to be considered. These include mechanistic comprehension of their sensitisation properties, the level of confidence of existing NAMs to cover microorganism-induced sensitisation, the definition of the applicability domains of relevant NAMs, and the need for novel methods to close toxicity data gaps.

The potential use of NAMs is described below as well as summarised in Table 1. The information is structured according to in vitro methods that address outcomes related to specific KEs in the skin and respiratory sensitisation processes.

**Table 1:** NAMs (in vitro and in silico methods) for elucidating the mechanisms of allergens and biological key events. The use and adaptation for skin and respiratory sensitisation assessment of microbial pesticides are still to be validated

Method	Description	Observations	References
*In vitro methods*			
EpiSensA (Epidermal Sensitisation Assay)	RHE-based assay that uses gene markers for evaluating the skin sensitization potential; it has the ability to evaluate more lipophilic compounds and has some metabolic capacity, so pre- and pro-haptens can be assessed	OECD-validated method (442D) for chemicals; KE 2	Saito et al. (2017)
SENS-IS assay	RHE-based assay that also employs gene markers for sensitisation as well as irritancy. It was shown to be applicable to a wide variety of materials, including highly lipophilic compounds and finished products	OECD Draft Guideline (2024); KEs 1, 2, 3	Cottrez et al. (2015, 2016, 2020)
h-CLAT (human cell line activation test)	Usually employed for LMW chemicals, but its applicability for evaluating the skin sensitisation of biologics and proteins has recently been explored	OECD-validated method (442E) for chemicals; KE 3	OECD 442E (2024); Tsukumo et al. (2018); Kobayashi-Tsukumo et al. (2019); Soltani et al. (2022)
U-SENS™ (U937 Cell Line Activation Test)	Quantifies the change in the expression of the cell surface marker CD86 in the human histiocytic lymphoma cell line U937 for supporting the discrimination between skin sensitisers and non-sensitisers. Pre-haptens (*i.e.* substances activated by oxidation) or pro-haptens (*i.e.* substances requiring enzymatic activation) were correctly predicted by this assay	OECD-validated method (442E) for chemicals; KE 3	OECD 442E (2024)
IL-8 Luc assay (Interleukin-8 Reporter Gene Assay)	Quantifies changes in IL-8 expression (luminescence-based assay, luciferase) in the THP-1-derived IL-8 reporter cell line to assess skin sensitisation. The IL-8 Luc assay can respond to a variety of stimuli other than haptens, such as bacterial toxins, Gram-positive and negative bacteria, and detergents	OECD-validated method (442E) for chemicals; KE 3	OECD 442E (2024); Kimura et al. (2020)
GARD™skin (Genomic Allergen Rapid Detection for Assessment of Skin Sensitisers)	Myeloid cell-based method employing MUTZ-3 cell line and genomic biomarker signature to assess KE3 of the AOP for skin sensitisation	OECD-validated method (442E) for chemicals; KE 3	OECD 442E (2024)
ALIsens®	A co-culture model with bronchial epithelial cells, endothelial cells and dendritic cells assembled in a 3D system capable of recapturing in vitro the physiological barrier. This model is used to evaluate barrier integrity, morphological changes, cytoprotective and cytokine responses, and viability, responding to various types of respiratory allergens	Non-OECD-adopted method; KE3	Chary et al. (2019); Burla et al. (2023)
GARD®air assay	Employs MUTZ-3 cell line and provides binary classification of respiratory sensitisers and non-sensitisers by expressing genomic biomarker signatures using machine learning approach. Its performance was evaluated for protein respiratory sensitisation and overall results demonstrated the detection of relevant events linked to type I hypersensitization	Non-OECD-adopted method; KE3	Zeller et al. (2018)
RBL assay (Rat Basophil Leukaemia cell assay)	In vitro assay with RBL-2H3 cell line, which, through IgE receptor binding and cross-linking by an antigen, cell mediators (e.g. ß-hexosaminidase) are released, and type I hypersensitivity events are recapture in vitro. It has demonstrated potential in identifying microbial respiratory sensitiser	Non-OECD-adopted method; KE5	Ward et al. (2018)
*In silico methods*			
AllerCatPro	Web tool that employs structural similarity analysis of both their amino acid sequences and 3D structures with known protein allergens to evaluate whether novel proteins have sequences similar to those associated with allergenic activity to predict protein allergenicity potential; accuracy of 84% was demonstrated	It can be applied to plant profilins, autoimmune allergens, nucleotide input sequences	Nguyen et al. (2022)
AlgPred	Web-based allergen tool that uses different approaches for the prediction of allergenic proteins (motif-based techniques, machine learning, and hybrid approach). AlgPred also predicts the potential IgE epitopes in the subjected hypothetical proteins (HPs)	It can be applied to HPs of *Alternaria alternata*, fungus involved in asthma development	Sundararaj et al. (2024)
Structural Database of Allergic Proteins (SDAP)	Predicts allergenic proteins by investigating the cross-reactivity between known allergens and potential allergens	Employed for HPs of fungal species *Alternaria alternata*	Sundararaj et al. (2024)
NetOGlyc 4.0	Model-based on genetic engineering approach using human cell lines for prediction of O‐glycosylation produces neural network predictions of mucin type GalNAc O-glycosylation sites in mammalian proteins	Used to predict glycosylation of fungal proteins accurately	Bamford et al. (2020)
Allergome database	Created to arrange the current knowledge on allergenic molecules in the most rational way		Abel-Fernández and Fernández-Caldas (2023)
WHO/IUIS ANSC database	Assigns official allergen names to purified natural or recombinant proteins that are characterised by standard methods of biochemistry and molecular biology and that bind IgE of individuals who are allergic to the source of the protein; provides up‐to‐date expert‐reviewed data on newly discovered allergens, including food allergens		Abel-Fernández and Fernández-Caldas (2023)
MEROPS database	Integrated source of information about peptidases, their substrates and inhibitors; uses a hierarchical classification: protein-species, family, clan, with an identifier at each level and contains sequences of characterised proteins from sequenced genomes of organisms of evolutionary, medical, or commercial significance	It can be applied to peptidase homologues from many bacterial and eukaryote proteomes	Krutz et al. (2020)

#### Bibliographic search, in silico tools and databases

In the absence of specific methods for regulatory purposes, a weight of evidence (WoE) approach has been proposed to support hazard classifications, such as for chemical respiratory sensitisers (Meek et al. 2023), Bibliographic research and evidence from multiple sources, including peer-reviewed publications and governmental reports, should be used to retrieve all information on the possible association with the microorganism or its products used as microbial pesticides, and any allergic effects reported in humans (Hardy et al. 2017). If data support the risk, then the information on sensitisation hazard should remain. An example of a WoE evaluation is the risk assessment of *Beauveria bassiana* strains, in which the sensitisation potential was based on evidence of allergenicity by inhalation in laboratory animals and humans as well as indications of allergic skin reactions in humans (Anastassiadou et al. 2020; Arena et al. 2017, 2018).

In recent years, several in silico predictions of the respiratory sensitisation potential of LMW chemicals have been developed based on the understanding of MIEs that lead to organ-level toxicity. Recent advancements in machine learning have enabled new possibilities for predicting respiratory sensitisers, utilising datasets of thousands of compounds classified as respiratory sensitisers or non-sensitisers based on structural characteristics, peer-reviewed literature, mechanistic alerts for dermal sensitisation, and newly developed rules for respiratory sensitisers. Additional mechanistic approaches were also utilised, considering steric factors and parameters derived from frontier molecular orbitals with high accuracy, specificity and sensitivity. Thus, for the identification of respiratory sensitisers, even for chemicals, the combination with additional computational, *in chemico* or in vitro methods was necessary to increase confidence (Hargitai et al. 2024).

In silico models capable of predicting cross-reactivity between protein allergens can also contribute to assessing the sensitisation properties of microorganisms, taking into account the ability to distinguish between immunogenicity and allergenicity caused by proteins (Basketter and Kimber 2018). A few web tools and databases have been proposed for in silico predictions, considering properties such as the presence of linear and 3D epitopes (antigenic determinants), glycosylation status, enzymatic activity, and stability to proteolytic digestion (physicochemical properties)*.* AllerCatPro is a web tool that utilises structural similarity analysis to evaluate whether novel proteins have sequences similar to those associated with allergenic activity based on known protein allergens. Examples of its use include profilins, autoimmune allergens, low allergenic proteins, very large proteins and nucleotide input sequences (Nguyen et al. 2022). AlgPred and the Structural Database of Allergic Proteins (SDAP) have been used to identify potential allergens from hypothetical proteins of the fungal species *Alternaria alternata* (Sundararaj et al. 2024). NetOGlyc 4.0, a tool designed to predict sites of O-glycosylation on mammalian peptides, has also been shown to accurately predict areas of hyper-O-glycosylation in fungal proteins (Bamford et al. 2020). Databases on allergens (*e.g.* Allergome and WHO/IUIS allergen database) are available as information sources on sensitisation of proteins, and MEROPS, a peptidase database, can provide information on protein stability to proteolytic digestion (Abel-Fernández and Fernández-Caldas 2023; Krutz et al. 2020). However, in silico data alone is insufficient to identify a protein as an allergen, as there is no consensus on whether respiratory hypersensitivity depends on IgE-mediated mechanisms. In some cases of chemical-induced asthma, only a minority of patients display detectable IgE (Hargitai et al. 2024). Immunological and physical properties can contribute to the effector activity of allergens (Hazebrouck et al. 2022); therefore, other methods should also be employed to identify true protein allergens.

#### In vitro methods

In vitro methods can provide tools for elucidating the mechanisms of allergens and responding to key biological events (Martel et al. 2010). However, the use and adaptation of currently validated in vitro methods for microorganisms remain a challenge, especially due to the difficulty of their overgrowth behaviour in culture, which results in nutrient depletion in the media and impaired growth of mammalian cells (Nims and Price 2017). Thus, an inactivation step or the use of crude extracts may be required before testing on traditional cell cultures. As alternatives, advanced cell culture systems, such as reconstructed epithelium models (3D organotypic models), may overcome this limitation, as they employ air–liquid interface (ALI) cultures that allow for topical treatments. Unlike submerged culture, ALI culture systems can reduce the interaction between microorganisms and culture media, avoiding nutrient depletion and secretion of endotoxins that affect the viability of mammalian cells (Baldassi et al. 2021).

Furthermore, especially for respiratory sensitisation, 3D organotypic models can be combined with advanced in vitro exposure systems (*e.g.* Vitrocell®) designed to provide more reliable results than submerged exposure by more closely replicating human physiology and controlling the number of substances delivered to cells in ALI cultures. Promising applications have also been demonstrated for various types of nanomaterials and virus research (Braakhuis et al. 2023; Kohl et al. 2023), suggesting that ALI cultures combined with these innovative exposure systems may circumvent the barriers to testing microorganisms in vitro. In addition, defining microbial positive and negative controls will be crucial to demonstrate and validate the applicability of in vitro methods for testing the sensitisation of microorganisms.

##### Epithelial cell activation (KE2)

The activation of inflammatory signalling by epithelial cells through the increased secretion of proinflammatory mediators, which subsequently activate DCs, is a common biological response in both skin and respiratory sensitisation processes (Hargitai et al. 2024; OECD 2012).

The OECD has adopted three test methods to address KE2 on the AOP for skin sensitisation under TG 442D (OECD 2024). The KeratinoSens™ and LuSens test methods use immortalised adherent cell lines derived from human keratinocytes that stably harbour a luciferase reporter gene. In these methods, responses to skin sensitisers are obtained by upregulating ARE-Nrf2-dependent genes, with the readouts measured by luminescence intensity. Although they have demonstrated good overall performance in identifying skin sensitisers (OECD 2024), KeratinoSens™ and LuSens employ monolayer cultures, which may not be suitable for testing microorganisms for the aforementioned reasons.

On the other hand, 3D skin models may provide viable alternatives to address KE2 for skin sensitisation hazards of microorganisms due to their greater biological complexity. Their use has the undoubted advantage of providing the ability to test a broader range of materials, including highly lipophilic compounds and finished products, which are known to be incompatible with current *in chemico* and in vitro methods using 2D cultures for skin sensitisation. The Epidermal Sensitisation Assay (EpiSensA), recently incorporated into OECD TG 442D, and the SENS-IS, currently under review by the OECD, employ reconstructed human epidermis (RHE) and show promising potential for adaptation in skin sensitisation assessments of microorganisms. The EpiSensA is based on changes in the expression of four marker genes (*ATF3*, *IL-8*, *DNAJB4*, and *GCLM*) associated with keratinocyte activation (inflammatory or cytoprotective) and has demonstrated good accuracy in testing a broad set of chemicals (Saito et al. 2017) The SENS-IS is based on the analysis of the expression of a panel of 65 genes grouped into one gene set for irritancy and two (SENS-IS and ARE) for sensitisation (Cottrez et al. 2015, 2016, 2020). Therefore, the SENS-IS method can help discriminate skin sensitisers from irritants and may provide additional value beyond assessing the sensitisation of microorganisms as skin irritation data are required for the regulatory approval of microbial pesticide active substances and products in the US, and products in the EU (Wend et al. 2024).

The applicability of the 3D models has recently been confirmed by Ahmed (Ahmed et al. 2024) who tested human skin explants co-cultured with autologous peripheral blood mononuclear cells to predict cytokine-associated adverse responses, skin sensitisation, and immunotoxic properties following 3-day incubation. This interesting approach using biologicals demonstrated the model to be relevant for microbial pesticides, for which skin contact is possible, even though only therapeutic antibodies have been tested so far. With 13 out of 16 therapeutic monoclonal antibodies correctly predicted, the relevance of topical application for therapeutic monoclonal antibodies is questionable.

In contrast to skin sensitisation, no validated test methods currently exist for KE2 or any other KE related to respiratory sensitisation. KE2 in respiratory sensitisation involves the activation of DCs and type 2 innate lymphoid cells (ILC2) by alarmins (*i.e.* pro-Th2 and DC attracting cytokines) secreted by lung epithelial cells. Thymic stromal lymphopoietin (TSLP), IL-33, IL-25, and GM-CSF are alarmins associated with DC activation and the maintenance of Th2 responses, with elevated levels observed in asthmatic patients. However, for some alarmins, such as TSLP, the specific involvement in respiratory sensitisation remains uncertain and may vary depending on the type of allergen. Notably, IL-25 release is triggered by LPS and ovalbumin (a protein found in egg whites). The proposed methods to address KE2 in respiratory sensitisation are those based on 3D lung models cultured in ALI systems, as they mimic the physiological barrier and can be used to evaluate barrier integrity, morphological changes, cytoprotective and cytokine responses, and viability (Hargitai et al. 2024).

##### Activation of dendritic cells (KE3)

DCs are a heterogeneous population of APC with a fundamental role in initiating and regulating adaptive immune responses, which act via the modulation of T cell responses (Galbiati et al. 2020; Hargitai et al. 2024). The activation of DCs by allergens is common for both skin and respiratory sensitisation, and this event involves diverse phenotypical and functional changes in these cells, such as the expression of co-stimulatory molecules and the release of cytokines and chemokines required in the interplay between DCs and T cells (Chary 2019; Galbiati et al. 2020; Hargitai et al. 2024).

CD54 and CD86 are the most well-established cell surface markers, commonly quantified on THP-1 cells (monocyte cell line) as part of the human cell line activation test (h-CLAT), an OECD-adopted method (OECD 442E) usually employed in the assessment of LMW chemicals (ECVAM 2015) but its applicability for evaluating the skin sensitisation of biologics has recently been explored. Soltani et al. (2022) used the h-CLAT assay, amongst other in vitro methods, to investigate the skin toxicity of the bacteria-derived antimicrobials reuterin, microcin J25, pediocin PA-1, bactofencin A, and nisin Z. Whilst microcin J25 and reuterin showed no skin sensitisation at concentrations up to 100 μg/mL and 40 mg/mL, respectively, pediocin PA-1, bactofencin A, and nisin Z caused sensitisation at concentrations higher than 100 μg/mL upregulating both CD54 and CD86.

The h-CLAT has also been adapted to test naturally occurring proteins, demonstrating that h-CLAT can be used to assess protein allergenic potency (Kobayashi-Tsukumo et al. 2019; Tsukumo et al. 2018). To eliminate the potential masking effect of LPS, which may influence CD86 and CD54 expression levels, a method was developed to exclude the effects of LPS when evaluating protein-induced skin sensitisation. Using two inhibitors, the caspase-1 inhibitor YVAD, which mitigates LPS-induced CD54 increases, and polymyxin B (PMB), which binds to the toxic lipid part of LPS, a more accurate assessment of protein allergenicity was enabled (Kobayashi-Tsukumo et al. 2019). The ability to distinguish between allergenicity and immunogenicity remains a challenge.

Regarding skin sensitisation, other OECD-validated methods (442E) based on KE 3 are U-SENS™ (U937 Cell Line Activation Test), IL-8 Luc assay (Interleukin-8 Reporter Gene Assay) and GARD™ skin (Genomic Allergen Rapid Detection for Assessment of Skin Sensitisers). Amongst these, the IL-8 Luc assay has been demonstrated to respond to a variety of stimuli, including environmental contaminants, bacterial toxins and detergents, as well as Gram-positive and Gram-negative bacteria (Kimura et al. 2020).

The h-CLAT has been considered for assessing the potential for respiratory sensitisation (Hargitai et al. 2024). For chemicals, certain respiratory sensitisers can increase at least one of the two established markers in the h-CLAT assay (Burla et al. 2023). However, it has also been reported that THP-1 monocultures may not adequately respond to respiratory allergens because they do not express adequate basal levels of these markers (Chary 2019). Thus, the proposed co-culture model (ALIsens®) which comprises bronchial epithelial cells (*e.g.* A549), endothelial cells (*e.g.* EA.hy926) and THP-1 cells assembled in a 3D system, is suggested to be a most promising model, since it captures the cellular architecture of the complex in vivo alveolar barrier (Burla et al. 2023; Chary 2019) and creates a microenvironment that could have a fundamental role in directing DC activation and responding to various types of respiratory allergens (Burla et al. 2023; Chary 2019). With special regard to HMW allergens, ALIsens® presents important features (*e.g.* the presence of an alveolar type II epithelial barrier) that justify its use for testing these types of allergens, for which the mechanisms of action differ from those of chemical respiratory sensitisers. The results to date are promising, and the expression of OX40L is proposed as a marker for identifying HMW sensitisers. However, there is no clear definition yet for cytokine markers of HMW allergens since the release pattern varies amongst different forms of allergens. For example, purified natural birch pollen (Bet v1) releases MCP-1, IL-10, MIP-3α, CCL20 and IL-6, whereas house dust mite extract does not (Chary 2019). Overall, the higher cellular complexity provided by this model, along with its good applicability domain for protein allergens, underscores its potential for assessing the sensitisation hazard of microorganisms.

The GARD™ air assay is another myeloid cell-based method proposed to assess potential respiratory sensitisers, which employs the MUTZ-3 cell line and provides binary classification of respiratory sensitisers and non-sensitisers by expressing genomic biomarker signatures using a machine learning approach. Besides the ring trial, which demonstrated high specificity and transferability for chemical respiratory sensitisation assessment, the performance of the GARD®air assay has also been evaluated for protein respiratory sensitisation (Zeller et al. 2018). This modified GARD®air assay identified 391 potential biomarkers as a predictive signature for protein allergens, and the overall results demonstrated that this in vitro method can capture relevant events linked to type I hypersensitisation (Zeller et al. 2018). However, adapting this assay to assess microorganism sensitisation presents challenges, and 3D models in ALI systems would be preferable.

##### Activation and proliferation of T cells (KE4) and B cell isotype switching (KE5)

The antigen-driven activation of T cells directs them to proliferate and differentiate into effector and memory populations. Depending on the nature of the allergens and the target organ, differentiation can occur toward Th1- and Th2-cell phenotypes (Hargitai et al. 2024; Thá et al. 2021). Respiratory sensitisers preferentially elicit a Th2 response, whereas skin sensitisers promote Th1-type reactions (Hargitai et al. 2024). However, specific food proteins can promote Th2-dependent sensitisation through the skin (Janssen et al. 2024). In this type of sensitisation, the production of antigen-specific IgE by B cells is crucial in provoking a strong inflammatory response upon subsequent exposure to the same allergen, consequently manifesting the clinical symptoms of allergies (Thá et al. 2021).

For skin sensitisation, the guinea pig methods (GPMT and Buehler test) and murine tests (LLNA and its three non-radioactive modifications) have regulatory acceptance for evaluating skin sensitisation, in response to KE4. These are in vivo methods and should be used as a last resort, in concordance with regulatory initiatives on the 3Rs. Furthermore, these methods were specifically designed to identify the skin sensitisation potential of LMW chemicals and may not accurately predict the sensitisation potential of proteins as their allergenicity would be entirely confounded by immunogenicity (Basketter and Kimber 2022). With respect to microorganisms in particular, topical applications of the test material to the skin (as per the protocol procedure in the Buehler test and LLNA) may raise uncertainties regarding negative responses, as microorganisms cannot penetrate intact skin due to their molecular size (Wend et al. 2024).

The LLNA has been adapted to assess potential respiratory sensitisers. In this adapted version of LLNA, known as the respiratory LLNA, mice are exposed by inhalation (head/nose-only) to the test material for three consecutive days, and different cytokine profiles are used to discriminate between contact allergens and respiratory allergens (Arts et al. 2008; Hargitai et al. 2024; Thá et al. 2021). However, for proteins, the problem of distinguishing their allergenicity from immunogenicity would remain, and thus, this would not be feasible to consider when assessing the sensitisation potential of HMW, as well as that of microorganisms, due to the similarity in nature of their secretory molecules.

Few non-animal alternatives have been proposed to date to address KE4. Peripheral blood mononuclear cells (PBMC)-based assays, utilising readouts of cell proliferation, cell surface markers and cytokine secretion, have been proposed (Aparicio-Soto et al. 2021). In particular, activation-induced surface marker assays (AIM assays) represent a novel option for a fast, comprehensive, and quantitative analysis of antigen-specific T cells (Aparicio-Soto et al. 2021). The AIM assay has already been successfully applied to detect 2,4,6-trinitrobenzenesulfonic acid (TNBS)-specific activated CD154 + CD4 + and CD137 + CD8 + T cells (Curato et al. 2022). As this is a short-term assay that can be performed at 5 and/or 16 h, it could be easily adapted for testing the sensitisation potential of microorganisms. The short duration of the assay would likely prevent the major challenges associated with handling microorganisms within mammalian cell cultures.

An IL-4-based 3D co-culture assay has also been proposed to address KE4 and to help discriminate between chemical respiratory and skin sensitisers. The assay consists of a two-step DC/T cell co-culture system in which peripheral allogeneic naïve CD4 + T cells are added to the DC co-culture system shortly after the activation of DCs by the chemical sensitiser, aiming to mimic the migration of DCs to draining lymph nodes. In this DC/T cell co-culture system, respiratory sensitisers but not skin sensitisers increased the mRNA expression of IL-4 in T cells (Mizoguchi et al. 2023). Although only evaluated for chemicals, this assay also has the potential to be applied to microorganisms due to the advantages of using ALI systems and models with higher complexity, as mentioned above.

As proteins can be related to the sensitisation potential of microorganisms and the production of IgE, antibodies is an important indicator of their allergenicity (Kimber et al. 2018; Loprieno 2012; OECD 2023a). Basophils have also been proposed for the study of respiratory sensitisation to proteins and microbial pesticides (OECD 2023a; Zeller et al. 2018). The Rat Basophil Leukaemia (RBL) cell assay employs the RBL-2H3 cell line (expressing the high-affinity IgE receptor (FcεRI)), which, through IgE receptor binding and cross-linking by an antigen, cell mediators (*e.g.* ß-hexosaminidase) are released, recapturing in vitro type I hypersensitivity events (Ward and Copeland 2018). Fungal microbes have been tested using the RBL assay, which has demonstrated potential for identifying microbial respiratory sensitisers by providing an index of functional IgE induction. Conversely, recent results with microbial pesticides indicate the need to define adequate controls, determine thresholds for allergy induction, and evaluate reproducibility and transferability to provide accurate respiratory responses to microbial respiratory sensitisation (OECD 2023a).

### Conclusion and perspectives

According to the precautionary principle, the Data Requirements of Regulation (EU) 283/2013 and the Uniform Principles of Regulation (EU) 546/2011, all microorganisms must be considered potential sensitisers until validated tests for sensitisation become available. Whilst no specific studies are currently required, in the EU, products containing microorganisms must include a warning phrase indicating their potential to cause sensitisation. This warning does not imply that the microorganisms are sensitisers but acknowledges their potential. Therefore, developing methods for the proper identification of skin and respiratory sensitisers in microbial pesticides is crucial to addressing their sensitisation potential effectively. Understanding the features and events causally and quantitatively related to the acquisition of microorganism sensitisation and allergy may help define new assessment methods (Krutz et al. 2020). Whilst additional adaptation and validation are needed to prove the applicability of current validated in vitro methods to microbial pesticides and their formulations, one can envision a multilevel strategy to be tailored to the nature of the microbial pesticide, starting with the assessment of dermal or inhalation exposure and absorption of the active substance and moving on to in vitro assessment of cell activation. It can be anticipated that the h-CLAT or any dendritic cell-based assay may be overly sensitive to the stimulation of microbial pesticides, as endotoxins or lipopolysaccharides can activate these cells. Therefore, caution should be taken when using DC-based methods. Some of the in vitro methods developed to assess skin sensitisation have been proven to work for mixtures, including agrochemical formulations (Cottrez et al. 2020; Settivari et al. 2015). Testing strategies, such as DAs anchored to human biology and mechanistic information, provide a promising approach to assessing the sensitisation potential of agrochemical formulations (Strickland et al., 2022), which should be extended to biopesticides.
